# Molecular Taxonomic Evidence for Two Distinct Genotypes of *Mycobacterium yongonense* via Genome-Based Phylogenetic Analysis

**DOI:** 10.1371/journal.pone.0152703

**Published:** 2016-03-31

**Authors:** Byoung-Jun Kim, Bo-Ram Kim, So-Young Lee, Ga-Na Kim, Yoon-Hoh Kook, Bum-Joon Kim

**Affiliations:** Department of Biomedical Sciences, Microbiology and Immunology, Cancer Research Institute, Institute of Endemic Diseases, and Seoul National University Medical Research Center (SNUMRC), Seoul National University College of Medicine, Seoul, Republic of Korea; Hellas, GREECE

## Abstract

Recently, we introduced a distinct *Mycobacterium intracellulare* INT-5 genotype, distantly related to other genotypes of *M*. *intracellulare* (INT-1 to -4). The aim of this study is to determine the exact taxonomic status of the *M*. *intracellulare* INT-5 genotype via genome-based phylogenetic analysis. To this end, genome sequences of the two INT-5 strains, MOTT-H4Y and MOTT-36Y were compared with *M*. *intracellulare* ATCC 13950^T^ and *Mycobacterium yongonense* DSM 45126^T^. Our phylogenetic analysis based on complete genome sequences, multi-locus sequence typing (MLST) of 35 target genes, and single nucleotide polymorphism (SNP) analysis indicated that the two INT-5 strains were more closely related to *M*. *yongonense* DSM 45126^T^ than the *M*. *intracellulare* strains. These results suggest their taxonomic transfer from *M*. *intracellulare* into *M*. *yongonense*. Finally, we selected 5 target genes (*argH*, *dnaA*, *deaD*, *hsp65*, and *recF*) and used SNPs for the identification of *M*. *yongonese* strains from other *M*. *avium* complex (MAC) strains. The application of the SNP analysis to 14 MAC clinical isolates enabled the selective identification of 4 *M*. *yongonense* clinical isolates from the other MACs. In conclusion, our genome-based phylogenetic analysis showed that the taxonomic status of two INT-5 strains, MOTT-H4Y and MOTT-36Y should be revised into *M*. *yongonense*. Our results also suggest that *M*. *yongonense* could be divided into 2 distinct genotypes (the Type I genotype with the *M*. *parascrofulaceum rpoB* gene and the Type II genotype with the *M*. *intracellulare rpoB* gene) depending on the presence of the lateral gene transfer of *rpoB* from *M*. *parascrofulaceum*.

## Introduction

Members of the *Mycobacterium avium* complex (MAC) are the most important nontuberculous mycobacteria (NTM) in terms of clinical and epidemiological aspects [1]. Traditionally, MAC includes two species: *M*. *avium* and *M*. *intracellulare* [2–4]. In addition to these 2 species, recent advances in molecular taxonomy have fueled the identification of novel species within the MAC [5–10]. Our group introduced a novel species *Mycobacterium yongonense*, which was closely related to *M*. *intracellulare*, from a Korean patient with pulmonary symptoms [11]. Notably, *M*. *yongonense* possessed a distinct RNA polymerase gene (*rpoB*) sequence that was identical to *M*. *parascrofulaceum*, which is a distantly related scotochromogen, suggesting the acquisition of the *rpoB* gene via a potential lateral gene transfer (LGT) event [12, 13]. Recently, *M*. *yongonense* strains causing pulmonary disease were also isolated from patients in Italy [14]. However, it should be noted that these strains harbored *rpoB* sequences that were almost identical to *M*. *intracellulare* and not *M*. *parascrofulaceum*, suggesting the possibility of the existence of another group of *M*. *yongonense* strains.

Our group reported that *M*. *intracellulare*-related strains from Korean patients showed genetic diversity. This diversity could be used to divide the strains into a total of five distinct groups (INT-1 to -5) via the molecular taxonomic approach using three independent chronometer molecules: *hsp65*, the internal transcribed spacer 1 (ITS-1) region and the 16S rRNA gene [15]. Of these genotypes, the INT-5 strains were distantly related to other genotypes of *M*. *intracellulare* (INT-1 to -4). We also introduced the complete genome sequences of two INT-5 strains, MOTT-H4Y and MOTT-36Y [16, 17], showing that they were more closely related to the genome of *M*. *yongonense* DSM 45126^T^ than *M*. *intracellulare* ATCC 13950^T^, despite they have *rpoB* sequences identical to *M*. *intracellulare*, but not *M*. *parascrofulaceum*. Furthermore, our recent study indicated that they harbored a novel insertion element (IS) sequence (IS*Myo2*) specific to *M*. *yongonense* [18]. Collectively, it suggests that MOTT-H4Y and MOTT-36Y might be variants of *M*. *yongonense* that were not subject to the *rpoB* LGT event from *M*. *parascrofulaceum*. Recently, it has been also reported that *M*. *yongonense* may be misidentified as one of the *M*. *intracellulare* strains [14, 19]. Therefore, the establishment of consensus guidelines is needed for the exact species delineation between *M*. *intracellulare* and *M*. *yongonense*.

So, the aim of the current study is to determine the exact taxonomic status of the two INT-5 strains, MOTT-H4Y and MOTT-36Y with the *M*. *intracellulare rpoB* sequences but with genomic sequences closely related to *M*. *yongonense* via genome-based phylogenetic analysis.

## Materials and Methods

### Mycobacterial Strains

A total of 16 clinical isolates were used in this study. These clinical isolates were collected from the Asan Medical Center (Seoul, Republic of Korea) and Seoul National University Hospital (Division of Pulmonary & Critical Care Medicine, Seoul, Republic of Korea). These strains were identified into genotypes by phylogenetic analysis based on *hsp65*, ITS1 and 16S rRNA gene sequences [15] and *rpoB* sequence analysis [6, 20] (S1 Table). These strains were grouped [15] as follows: five INT-1 strains (Asan 29591, 29778, 36309, 37128, and 37721), five INT-2 strains (Asan 36638, 37016, 38392, 38402, and 38585), and six INT-5 strains (Asan 36527 and 36912, MOTT-68Y, MOTT-H4Y, MOTT-36Y and Rhu). For genomic DNA extraction, the clinical isolates were cultured on Middlebrook 7H10 agar plates supplemented with OADC (BD GmbH, Heidelberg, Germany) for 7–10 days in a 5% CO_2_ incubator at 37°C. Genomic DNA was prepared by the bead beater-phenol extraction method as previously described [21].

### Complete Genome Sequence-Based Phylogenetic Analysis

For the phylogenetic analysis of the two INT-5 strains (MOTT-H4Y and MOTT-36Y), their genome sequences [MOTT-H4Y (Genbank accession No. AKIG00000000) and MOTT-36Y (Genbank accession No. NC_017904)] [16, 17] were compared with those of *M*. *yongonense* DSM 45126^T^ (Genbank accession No. NC_021715) [13], *M*. *parascrofulaceum* ATCC BAA-614^T^ (Genbank accession No. ADNV00000000), and 3 strains belonging to *M*. *intracellulare* [one INT-1 strain: *M*. *intracellulare* MOTT-64 (GenBank accession No. NC_016948) and two INT-2 strains: *M*. *intracellulare* ATCC 13950^T^ (GenBank accession No. NC_016946) and *M*. *intracellulare* MOTT-02 (GenBank accession No. NC_016947)] [22–24]. These genome sequences were subjected to whole-genome multiple sequence alignments using the neighbor-joining method [25] by the Mauve Genome Alignments software (http://darlinglab.org/mauve/mauve.html). A phylogenetic tree was generated using the aligned genome sequences and visualized by the TreeViewX program (http://darwin.zoology.gla.ac.uk/~rpage/treeviewx/); additionally, a Venn diagram was constructed by the BLASTCLUST program. The minimum length coverage and identity threshold in BLASTCLUST were 0.9 and 95%, respectively.

### Phylogenetic Analysis Based on *rpoB* and 35 Selected Target Gene Sequences or Single Nucleotide Polymorphisms (SNPs) of the 35 Selected Target Gene Sequences

To analyze the sequence differences among the 3 *M*. *intracellulare* strains (*M*. *intracellulare* ATCC 13950^T^, *M*. *intracellulare* MOTT-02, and *M*. *intracellulare* MOTT-64), 2 INT-5 strains (MOTT-H4Y and MOTT-36Y) and *M*. *yongonense* DSM 45126^T^, the *rpoB* gene and 35 additional gene sequences were selected from the genome sequences. In the selected 35 genes, 10 genes (*argH*, *cya*, *glpK*, *hsp65*, *murC*, *pta*, *recA*, *secA1* and *sodA*) [26–29] were included for mycobacterial MLST analysis, and other 25 genes were were randomly selected in the housekeeping genes without any standards. The list of chosen genes is as follows: adenylate kinase (*adk*), argininosuccinate lyase (*argH*), chorismate synthase (*aroC*), shikimate 5-dehydrogenase (*aroE*), F0F1 ATP synthase subunit beta (*atpD*), adenylate cyclase (*cya*), cytochrome b6 (*cytB*), ATP-dependent RNA helicase, dead/death box family protein (*deaD*), chromosomal replication initiation protein (*dnaA*), DNA primase (*dnaG*), molecular chaperone DnaK (*dnaK*), chaperone protein (*dnaJ*), 3-oxoacyl-(acyl-carrier-protein) reductase (*fabG*), cell division protein FtsZ (*ftsZ*), fumarate hydratase (*fumC*), malate synthase G (*glcB*), glutamine synthetase type I (*glnA*), glycerol kinase (*glpK*), fructose 1,6-bisphosphatase II (*glpX*), 6-phosphogluconate dehydrogenase (*gnd*), DNA gyrase subunit B (*gyrB*), heat-shock protein 65 kD (*hsp65*), myo-inositol-1-phosphate synthase (*ino1*), NAD-dependent DNA ligase LigA (*ligA*), ATP-dependent DNA ligase (*ligB* and *ligC*), UDP-N-acetylmuramate-L-alanine ligase (*murC*), endonuclease III (*nth*), glucose-6-phosphate isomerase (*pgi*), phosphoglycerate kinase (*pgk*), phosphate acetyltransferase (*pta*), recombinase A (*recA*), recombination protein F (*recF*), preprotein translocase subunit SecA (*secA1*), and [Mn]-superoxide dismutase (*sodA*) (Table 1). The retrieved *rpoB* gene or the concatenated 35 selected gene sequences were multiply aligned using the ClustalW method in the MEGA 4.0 software [30]. Using the multiple alignment matrix, phylogenetic trees were constructed using the neighbor-joining method [25] in the MEGA 4.0 software [30]. The bootstrap values were calculated from 1,000 replications.

**Table 1:** Total and *M*. *yongonense*-group related-SNPs from targeted 35 genes of *Mycobacterium intracellulare* strains.

No.	Genes	Compared nucleotide size (bp)	Total SNPs (n)	*M*. *yongonense*-group related-SNPs (n)
1	*adk*	534	49	0
2	*argH*	1,431	199	10
3	*aroC*	1,206	134	0
4	*aroE*	888	134	0
5	*atpD*	1,461	164	0
6	*cya*	1,554	207	5
7	*cytB*	1,704	185	0
8	*deaD*	1,704	228	14
9	*dnaA*	1,503	193	11
10	*dnaG*	1,953	250	5
11	*dnaJ*	1,149	124	2
12	*dnaK*	1,860	118	2
13	*fabG*	768	80	0
14	*ftsZ*	1,161	108	0
15	*fumC*	1,407	152	0
16	*glcB*	2,169	183	0
17	*glnA*	1,437	106	1
18	*glpK*	1,527	223	2
19	*glpX*	987	101	0
20	*gnd*	1,521	147	0
21	*gyrB*	2,034	245	4
22	*hsp65*	1,626	103	6
23	*ino1*	1,095	81	2
24	*ligA*	2,082	257	3
25	*ligB*	1,530	167	0
26	*ligC*	1,056	173	3
27	*murC*	1,479	207	0
28	*nth*	792	84	0
29	*pgi*	1,665	203	0
30	*pgk*	1,236	187	0
31	*pta*	2,160	328	3
32	*recA*	1,053	49	0
33	*recF*	1,158	235	18
34	*rpoB*	3,390	185	0
35	*secA1*	2,829	268	3
36	*sodA*	624	118	0

SNPs were extracted from the multiple alignments of *rpoB* gene sequences and the 35 selected gene sequences from the 3 *M*. *intracellulare* strains (*M*. *intracellulare* ATCC 13950^T^, *M*. *intracellulare* MOTT-02, and *M*. *intracellulare* MOTT-64), 2 *M*. *intracellulare* INT-5 strains (MOTT-H4Y and MOTT-36Y), *M*. *yongonense* DSM 45126^T^ and *M*. *parascrofulaceum* ATCC BAA-614^T^. Then, the patterns were compared. Additionally, the extracted SNP sequences were concatenated and used to construct a phylogenetic tree as described above.

### Application of SNP Analysis to MAC Clinical Isolates

To confirm the different SNP patterns between the INT-5 strains and other *M*. *intracellulare* strains (INT-1 or INT-2 strains), five genes of other *M*. *intracellulare* clinical isolates, which proved to have more *M*. *yongonense*-group related signature SNPs than others, were amplified and sequenced for further analysis. The five selected genes were *argH*, *dnaA*, *deaD*, *hsp65* and *recF*. Genomic DNA from each of the five INT-1 strains (Asan 29591, 29778, 36309, 37128, and 37721), five INT-2 strains (Asan 36638, 37016, 38392, 38402, and 38585), and four INT-5 strains (Asan 36527 and 36912, MOTT-68Y and Rhu) was used to amplify the five selected genes. As a positive and a negative control of PCR of 5 genes, genomic DNA of *M*. *intracellulare* ATCC 13950^T^ and distilled water were also used. The five primer sets were as follows: *argH*, argH_F 5’–TGA GCA AGT CCA CCC ATT TC– 3’ and argH_R 5’–TGG CGT CGA TGG AGT TGT C– 3’; *dnaA*, dnaA_F 5’–ACG AGC CTC AAC CGC C– 3’ and dnaA_R 5’–CTC ACG GCA CAG GTA CAT CG–R’; *deaD*, DEAD_F 5’–GGA ATA CAA GCA GGT GGC ACT– 3’ and DEAD_R 5’–GCG TTC GTA GTC CTG GAC CA– 3’; *hsp65*, hspF3 5’–ATC GCC AAG GAG ATC GAG CT– 3’ and hspR4 5’–AAG GTG CCG CGG ATC TTG TT– 3’ and *recF*, recF_F 5’–GAA ATC CCT GTC TGG CGC– 3’ and recF_R 5’–TCA TGC GCG CAT CTC C– 3’. Template DNA and each primer pair (20 pmol) were added to the PCR premix (AccuPower PCR PreMix, Bioneer), and PCR was conducted by subjecting the samples to 5 min at 95°C, followed by 30 cycles of 95°C for 30 s, 58–60°C for 30 s, and 72°C for 1 min, and a final extension at 72°C for 5 min. The PCR reaction was performed in a MyCycler (Bio-RAD). The PCR products were detected and purified using the MEGAquick-Spin Total Fragment DNA Purification kit (iNtRON) for direct sequencing. Sequencing reactions were performed using an MJ Research PTC-225 Peltier Thermal Cycler and ABI PRISM BigDye Terminator Cycle Sequencing kits with the AmpliTaq DNA polymerase (FS enzyme, Applied Biosystems) following the manufacturer’s protocols. The obtained sequences were aligned using the MegAlign software package (DNASTAR), and then the SNPs in the sequences were analyzed.

## Results

### Genome Sequence-Based Phylogenetic Analysis of Two INT-5 Strains, MOTT-H4Y and MOTT-36Y

**Fig 1:**
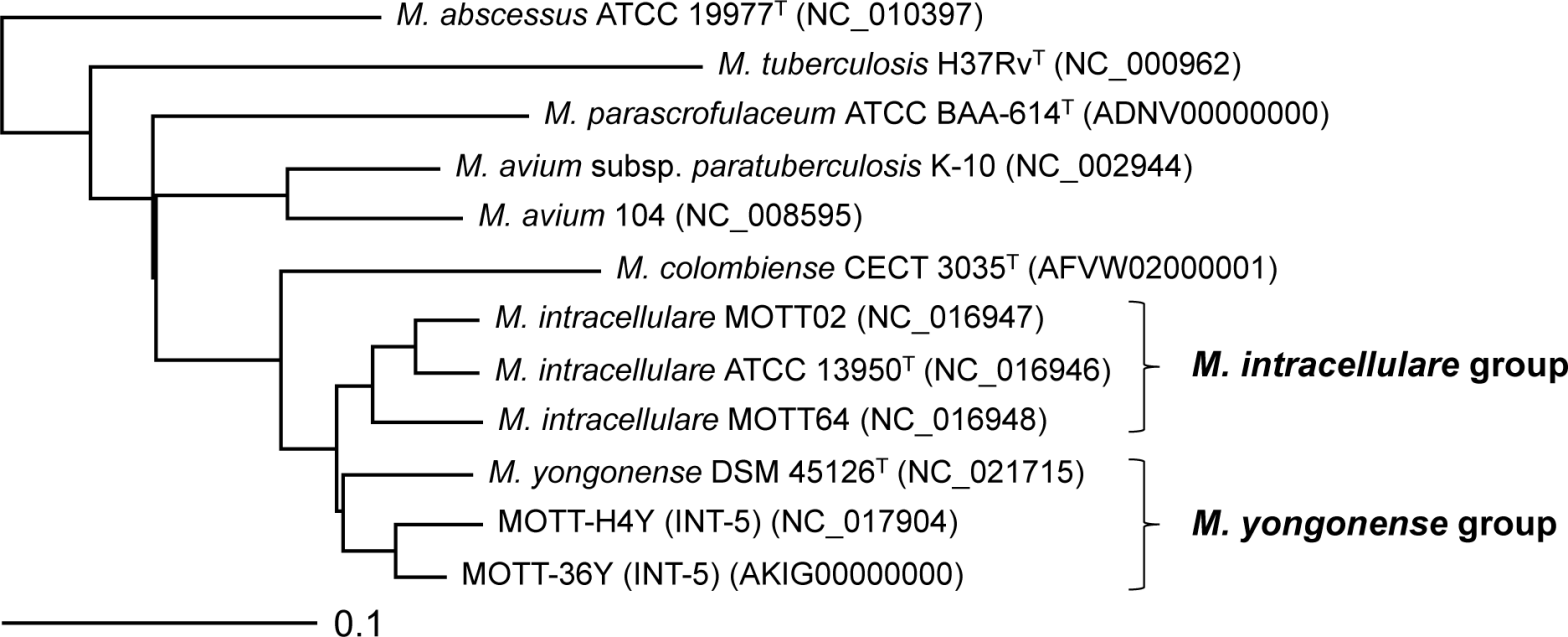
Phylogenetic tree based on whole-genome sequences of 3 *Mycobacterium intracellulare* group, 2 INT-5 group, *M*. *yongonense* and other mycobacterial strains. The tree was calculated using the neighbor-joining method by the Mauve Genome Alignment software and visualized by the TreeViewX program. The bar indicates the number of substitutions per nucleotide position.

The phylogenetic relationships between 3 *M*. *intracellulare* strains (ATCC 13950^T^, MOTT-02, and MOTT-64), 2 INT-5 strains (MOTT-H4Y and MOTT-36Y) and one *M*. *yongonense* strain (DSM 45126^T^) were analyzed using the genome sequence information (Fig 1). All 6 strains were clustered together in a branch. The strains were separated into two different branches: one including 3 *M*. *intracellulare* strains (ATCC 13950^T^, MOTT-02, and MOTT-64) and the other including *M*. *yongonense* DSM 45126^T^ and the two INT-5 strains, MOTT-H4Y and MOTT-36Y. This result indicated that the two INT-5 strains were more closely related to *M*. *yongonense* DSM 45126^T^ than the 3 *M*. *intracellulare* strains (ATCC 13950^T^, MOTT-02, and MOTT-64) (Fig 1).

**Fig 2:**
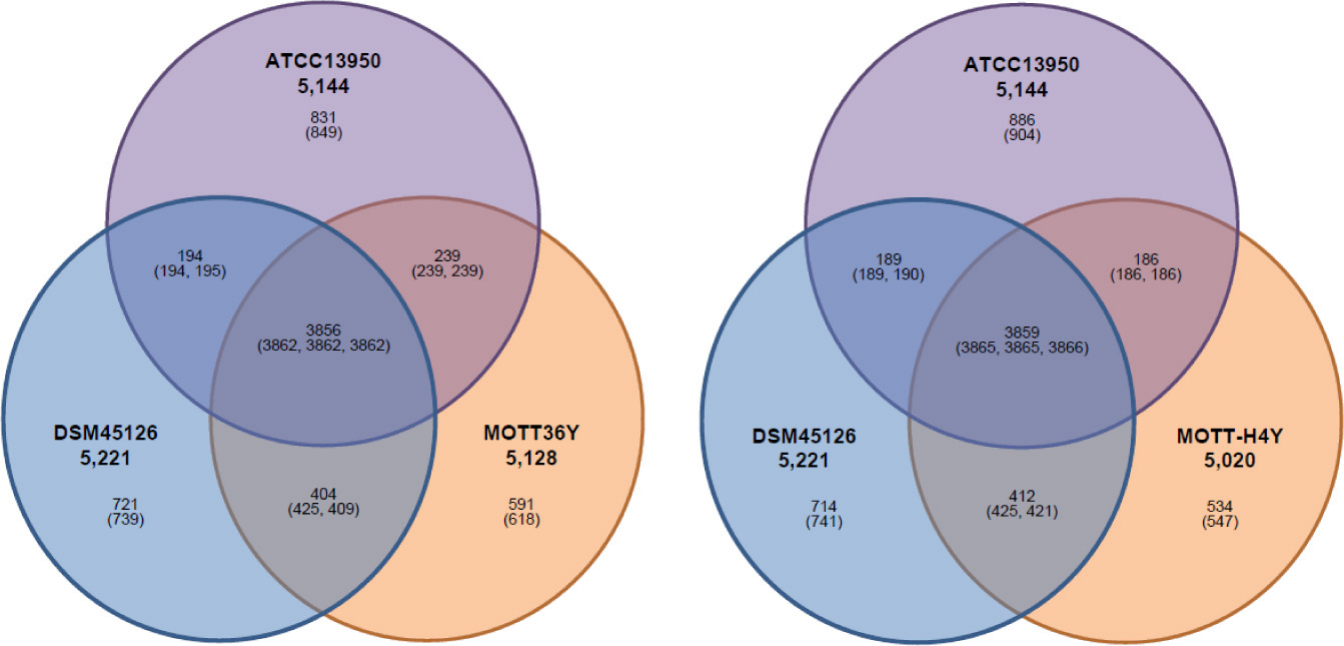
Venn diagrams based on genome information of INT-5 strains. Venn diagrams showing orthologous ORFs among four mycobacterial species as determined by BLASTCLUST analysis. Numbers in parenthesis include paralogous ORFs.

To assess the number of genes shared between each genome, we performed a BLASTCLUST analysis on the four genomes (*M*. *intracellulare* ATCC 13950^T^, MOTT-H4Y, and *M*. *yongonense* DSM 45126^T^ or *M*. *intracellulare* ATCC 13950^T^, MOTT-36Y, and *M*. *yongonense* DSM 45126^T^). At the level of 95% identity, *M*. *intracellulare* MOTT-36Y or MOTT-H4Y shared more orthologous coding sequences (CDSs) with *M*. *yongonense* DSM 45126^T^ (4,271/5,128 CDSs, 83.3% or 4,287/5,020 CDSs, 85.4%, respectively) than *M*. *intracellulare* ATCC 13950^T^ (4,101/5,128 CDSs, 80.0% or 4,052/5,020 CDSs, 80.7%, respectively) (Fig 2). This finding supported the results of our phylogenetic study that the two INT-5 strains might belong to *M*. *yongonense* rather than to *M*. *intracellulare* (Fig 1).

### Phylogenetic Analysis of Two INT-5 Strains, MOTT-H4Y and MOTT-36Y Based on the *rpoB* Gene Sequences and the Sequences of 35 Selected Target Genes

The taxonomic signature of *M*. *yongonense* was previously reported to be based on the *rpoB* gene sequence. The sequence of this gene is identical to the distantly related species *M*. *parascrofulaceum*, which enables the separation of the 2 closely related species *M*. *intracellulare* and *M*. *yongonense* [11, 12]. Therefore, to obtain the exact taxonomic delineation of the two INT-5 strains we compared their taxonomic location by phylogenetic analysis based on the sequences of *rpoB* and 35 selected target genes.

**Fig 3:**
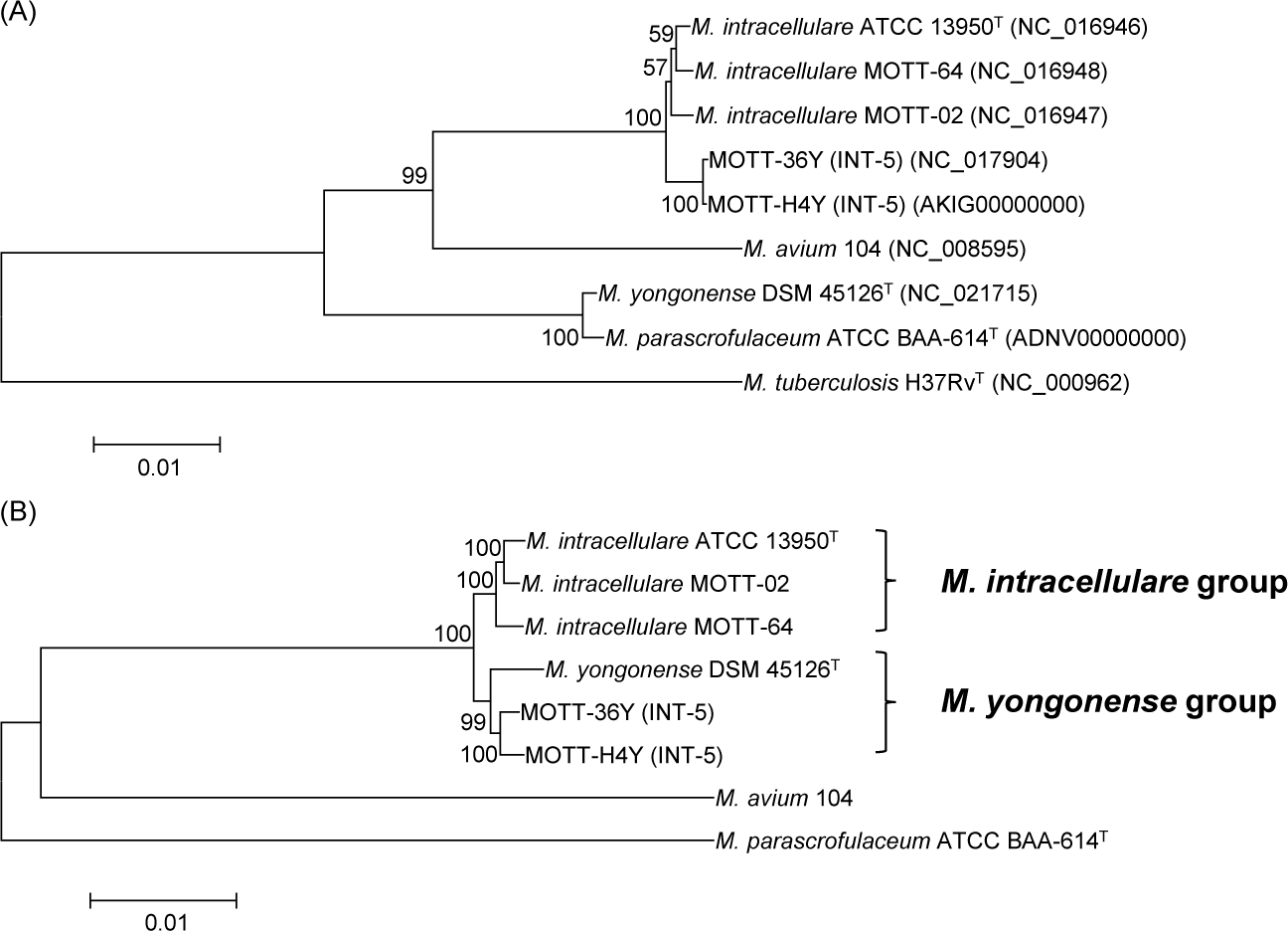
Neighbor-joining phylogenetic tree based on the *rpoB* or 35 concatenated genes from 6 *Mycobacterium intracellulare* strains. (A) A tree based on the whole *rpoB* gene sequences from 3 *M*. *intracellulare*, 2 INT-5 strains, *M*. *yongonense*, and *M*. *parascrofulaceum*. (B) A tree based on the 35 concatenated gene sequences from 3 *M*. *intracellulare*, 2 INT-5 strains, *M*. *yongonense*, and *M*. *parascrofulaceum*. The bootstrap values were calculated from 1,000 replications and values <50% were not shown. The bar indicates the number of substitutions per nucleotide position. *M*. *tuberculosis* H37Rv and *M*. *avium* 104 were used as outgroups in the *rpoB* gene- or concatenated genes-based phylogenetic trees, respectively.

The entire sequences of *rpoB* and the 35 selected genes were retrieved from the genome sequences of 6 mycobacterial strains [3 *M*. *intracellulare* strains (*M*. *intracellulare* ATCC 13950^T^, MOTT-02, and MOTT-64), 2 INT-5 strains (MOTT-36Y and MOTT-H4Y) and *M*. *yongonense* DSM 45126^T^] (Table 1) and subjected to phylogenetic analysis. In the *rpoB* gene (3,375 to 3,462 bp)-based phylogenetic analysis, the two INT-5 strains MOTT-H4Y and MOTT-36Y were clustered into the group including the *M*. *intracellulare* strains (*M*. *intracellulare* ATCC 13950^T^, MOTT-02, and MOTT-64) and were separated from *M*. *yongonense* DSM 45126^T^and *M*. *parascrofulaceum* ATCC BAA-614 (Fig 3A). However, in the phylogenetic analyses based on the sequences of the 35 selected genes, the two INT-5 strains MOTT-H4Y and MOTT-36Y were clustered into *M*. *yongonense* DSM 45126^T^ and separated from the other 3 *M*. *intracellulare* strains with a high bootstrap value (> 99%), as shown in the genome sequence-based phylogenetic analysis (Figs 1 and 3B). These results suggest that there may be a distinct *M*. *yongonense* genotype having an *rpoB* gene sequence that is almost identical to *M*. *intracellulare*.

### Phylogenetic Analysis of Two INT-5 Strains (MOTT-H4Y and MOTT-36Y) Based on Single Nucleotide Polymorphisms (SNPs) of the *rpoB* and 35 Targeted Genes

Multiple alignments of the *rpoB* and 35 gene sequences from the 3 *M*. *intracellualre* (*M*. *intracellulare*, MOTT-02 and MOTT-64), 2 INT-5 (MOTT-H4Y and MOTT-36Y), *M*. *yongonense* and *M*. *parascrofulaceum* showed that there were *M*. *yongonense* group- related SNPs in 17 genes [*hsp65* (6 *M*. *yongonense* group-related SNPs/103 total SNPs), *argH* (10/199), *cya* (5/207), *dnaJ* (2/124), *glpK* (2/223), *pta* (3/328), *recF* (18/235), *secA1* (3/268), *deaD* (14/228), *dnaA* (11/193), *dnaG* (5/250), *dnaK* (2/118), *glnA* (1/106), *gyrB* (4/245), *ino1* (2/81), *ligA* (3/257), and *ligC* (3/173)] (Table 1). Detailed *M*. *yongonense* group-related SNP signatures are listed in Table 2.

**Table 2:** Details of *M*. *yongonense* group-related SNP signatures.

Genes	*M*. *yongonense* group-related SNP signatures
*argH*	C105**T**^a^ C132**G** C138**G** C244**T** G303**A** C306**G** C322**T** G339**C** T566**C** C603**A**
*cya*	C399**G** C414**G** G432**C** C483**G** C504**T**
*deaD*	G204**C** G210**C** G216**T** G276**A** A315**T** C376**T** C648**T** G840**A** A894**G** T993**C** G1062**C** C1068**G** G1191**A** C1383**T**
*dnaA*	T222**C** C441**G** G639**A** G651**C** G714**C** G759**A** C921**T** C1035**G** G1080**A** A1326**G** G1341**C**
*dnaG*	C897**G** G921**T** C1350**G** C1488**T** G1560**T**
*dnaJ*	C849**T** C1008**T**
*dnaK*	C1476**T** T1509**C**
*glnA*	T1434**A**
*glpK*	T30**C** C723**G**
*gyrB*	C297**G** G375**C** C660**T** G702**C**
*hsp65*	G198**A** C555**G** G633**C** C726**T** G1191**C** G1539**C**
*ino1*	G291**C** G396**A**
*ligA*	A146**C** C441**G** G1986**A**
*ligC*	C384**T** G813**A** C933**T**
*pta*	C1368**T** C1371**T** C1464**T**
*recF*	C171**T** C249**A** C264**T** C279**T** G336**A** T429**C** A467**G** T534**G** G570**C** C579**T** G586**C** T660**C** G771**T** T796**C** T937**C** G963**A** C1009**T** G1123**A**
*secA1*	G645**C** T717**G** C1854**G**

All the nucleotide positions were determined from *Mycobacterium intracellulare* ATCC 13950^T^ strain.

^*a*^ Bold characters represent *M*. *yongonense* group-related SNPs.

**Fig 4:**
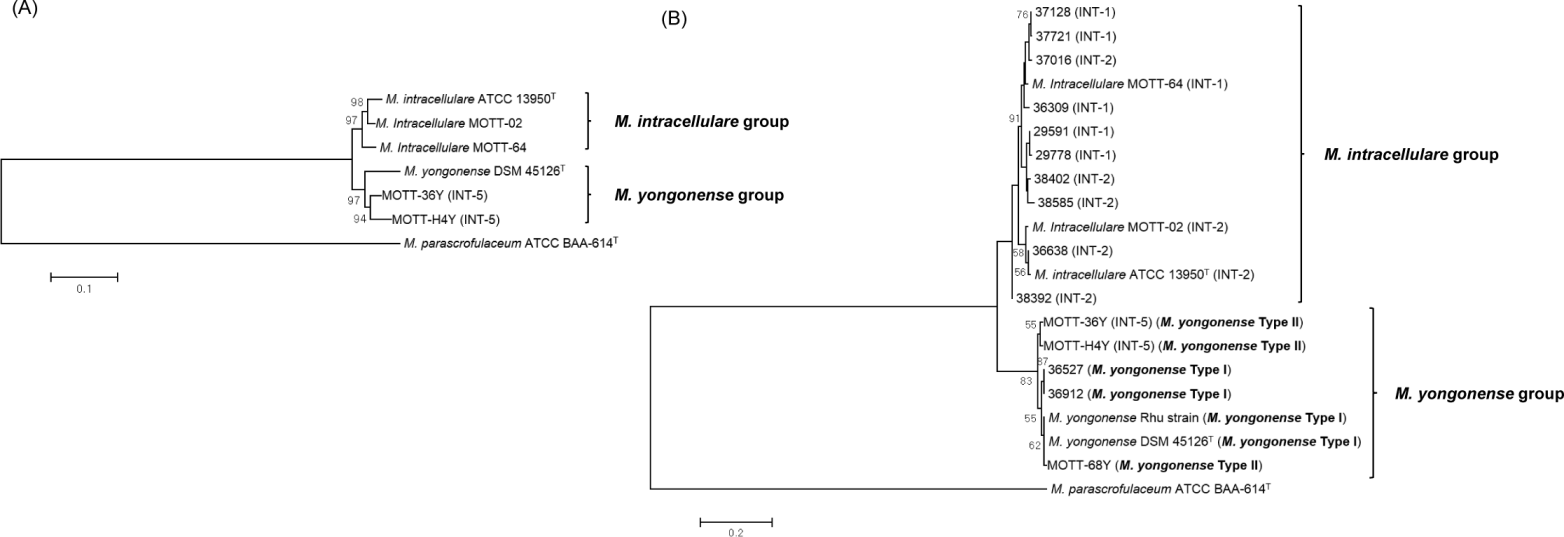
Neighbor-joining phylogenetic tree based on concatenated SNPs. (A) A concatenated SNP-based tree from 35 target genes of 3 *M*. *intracellulare*, 2 INT-5 strains, *M*. *yongonense*, and *M*. *parascrofulaceum*. (B) A concatenated SNP-based tree from 5 selected genes (*argH*, *dnaA*, *deaD*, *hsp65* and *recF*) from 3 *M*. *intracellulare*, 2 INT-5 strains, *M*. *yongonense*, and *M*. *parascrofulaceum* and 14 clinical isolate strains. The indicated INT-groupings were assigned in a previous report [15]. The bootstrap values were calculated from 1,000 replications and values <50% were not shown. The bar indicates the number of substitutions per nucleotide position.

In the case of *rpoB* gene, there was no *M*. *yongonense* group-related SNPs, however, *rpoB* gene of *M*. *yongonense* shared identical 151 SNPs with that of *M*. *parascrofulaceum*. A concatenated phylogenetic tree was constructed using the extracted SNP sequences. The tree showed that the two INT-5 strains were clustered into *M*. *yongonense* DSM 45126^T^ and separated from the other 3 *M*. *intracellulare* strains based on the phylogenetic analyses of the complete genome sequences and 35 concatenated gene sequences (Figs 1, 3B and 4A).

### Application of the *M*. *yongonense*-Related SNP Analysis to MAC Clinical Isolates

To develop SNP analysis to enable the selective identification of *M*. *yongonense* strains from the MAC strains, five genes (*argH*, *deaD*, *dnaA*, *hsp65* and *recF*) were selected that possessed a higher number of *M*. *yongonense* group (*M*. *yongonense* DSM 45126^T^ and two INT-5 strains MOTT-H4Y and MOTT-36Y) -related SNPs compared to the other genes. To explore the usefulness of this assay, sequence analysis of the five genes was applied to a total of 14 MAC clinical isolates from different Korean patients [five *M*. *intracellulare* INT-1 strains (Asan 29591, 29778, 36309, 37128 and 37721), five *M*. *intracellulare* INT-2 strains (Asan 36638, 37016, 38392, 38402 and 38585), and four INT-5 strains (Asan 36527, 36912, Rhu and MOTT-68Y)] and 7 was subjected to phylogenetic analysis.

All four INT-5 strains had 10 *M*. *yongonense* group-related SNPs in the partial *argH* gene sequence out of 10 *M*. *yongonense* group-related SNPs (from 105 nt to 657 nt). However, two INT-1 (Asan 29778 and 37721) and two INT-2 group (Asan 37016 and 38392) strains also shared one *M*. *yongonense* group-related SNP (G339C). All four INT-5 strains had 7 *M*. *yongonense* group-related SNPs in the partial *dnaA* gene sequence out of 7 *M*. *yongonense* group-related SNPs (from 627 nt to 1257 nt). However, one INT-2 group strain (Asan 38392) also shared one *M*. *yongonense* group-related SNP (C921T). All four INT-5 strains had 7 *M*. *yongonense* group-related SNPs in the partial *deaD* gene sequence out of 7 *M*. *yongonense* group-related SNPs (from 588 nt to 1191 nt). However, three INT-2 strains also shared one or four *M*. *yongonense* group-related SNPs (Asan 36638: C1068G; Asan 38392: C1068G; and Asan 38585: C648T, C681T, G1062C, and C1068G). In the partial *hsp65* gene sequence with 4 *M*. *yongonense* group-related SNPs (from 192 nt to 726 nt), two INT-5 group strains (Asan 36527 and Asan 36912) had only one *M*. *yongonense* group-related SNPs (G198A) and the three INT-1 or INT-2 SNPs (C555, G633 and C726), while the other two strains (Rhu and MOTT-68Y) had 4 *M*. *yongonense* group-related SNPs. All of the INT-5 group strains had 11 *M*. *yongonense* group-related SNPs in the partial *recF* gene sequence out of 11 *M*. *yongonense* group-related SNPs (from 520 nt and 1131 nt). However, one INT-1 (Asan 36309: T660C and G1123A) and one INT-2 strain (Asan 38392: T660C) shared one or two *M*. *yongonense* group-related SNPs.

The phylogenetic analysis based on the concatenated SNP sequences (395 bp) extracted from the five target genes showed that all four INT-5 strains of *M*. *yongonense* may share were clearly separated from the other *M*. *intracellulare* clinical isolates (Fig 4B). These results suggested the usefulness of SNP analysis for the taxonomic separation of *M*. *yongonense* from closely related *M*. *intracellulare* strains.

## Discussion

In the present study, our phylogenetic analysis based on complete genome sequences, multi-locus sequence typing (MLST) of 35 target genes, and single nucleotide polymorphism (SNP) analysis indicated that the two INT-5 strains, MOTT-H4Y and MOTT-36Y were more closely related to *M*. *yongonense* DSM 45126^T^ than the *M*. *intracellulare* strains. This finding suggests the presence of another distinct genotype in *M*. *yongonense* that may not have been subjected to the LGT event of *rpoB* from *M*. *parascrofulaceum*. Therefore, *M*. *yongonense* could be divided into 2 distinct genotypes: one with the *M*. *parascrofulaceum rpoB* gene and the other with the *M*. *intracellulare rpoB* gene, depending on the presence of the LGT event of *rpoB* from *M*. *parascrofulaceum* (Figs 1 and 3). Here, we proposed the former and the latter as the *M*. *yongonense* Type I and Type II genotypes, respectively.

To date, a total of 3 strains (*M*. *yongonense* DSM 45126^T^, Asan 36912 and Asan 36527) belonging to the *M*. *yongonense* Type I genotype have been introduced via our 2 recent reports [11, 12]. The Rhu strain used in this study was also identified as the Type I genotype by *rpoB* gene analysis (data not shown). In addition to MOTT-H4Y and MOTT-36Y, one additional strain (MOTT-68Y) used in this study was identified as the *M*. *yongonense* Type II genotype. Although detailed taxonomic proof is needed, the *M*. *yongonense* strains recently isolated in Italy have the potential to be included in the *M*. *yongonense* Type II genotype.

LGT is the major mechanism by which bacteria can acquire genetic diversity, guaranteeing their survival under harsh environmental conditions [31, 32]. However, it is generally accepted that mycobacteria are more resistant to LGT compared to other bacteria, possibly due to the unusually mycolic acid-rich cell wall structure and the relative scarcity of genetic elements such as plasmids and transposable elements [33–35]. Notably, because the *M*. *yongonense* strains were demonstrated to possess an *rpoB* gene that might have been laterally transferred from the distantly-related scotochromogenic species *M*. *parascrofulaceum*, these strains have gained increasing importance in the mycobacterial taxonomic fields. One of the noteworthy findings in this study is the identification of a novel genotype of *M*. *yongonense* without the *rpoB* gene from the LGT event in its genome. A genome comparison study between 3 mycobacterial groups [the *M*. *yongonense* Type I (subject to the LGT event) and Type II genotypes (without the LGT event) and *M*. *parascrofulaceum* (gene donor for LGT)] may provide novel insights into our understandings regarding mycobacterial LGT mechanisms.

In the present study, we developed an SNP analysis targeting 5 genes (*argH*, *deaD*, *dnaA*, *hsp65* and *recF*) for the separation of *M*. *yongonense* from the closely related *M*. *intracellulare* strains. The concatenated 395-bp SNP-based phylogenetic analysis clearly separated 7 *M*. *yongonense* strains from 12 closely related *M*. *intracellulare* strains belonging to the INT-I and INT-2 genotypes, which were the first and the second most prevalent genotypes in Korean patients infected with *M*. *intracellulare*, respectively, with 83% bootstrap values (Fig 4A). This result suggests the feasibility of this assay for the selective identification of *M*. *yongonense* strains in clinical settings. Interestingly, this assay could not differentiate 4 Type I (DSM 45126^T^, Asan36527, Asan 36912, and Rhu) and 3 Type II strains (MOTT-H4Y, MOTT-36Y and MOTT-68Y) (Fig 4B), suggesting the potential for gene exchanges by LGT events between the 2 genotypes. Notably, a total of 39 *M*. *yongonense* signature SNPs out of the 395 selected SNPs were found. These SNPs could be used for the development of *M*. *yongonense*-specific molecular diagnostic methods.

In conclusion, our genome-based phylogenetic analysis indicated that the taxonomic status of the two INT-5 strains, MOTT-H4Y and MOTT-36Y previously identified as *M*. *intracellulare* should be revised to *M*. *yongonense*. Taken together, *M*. *yongonense* could be divided into 2 distinct genotypes depending on the presence of the LGT event of *rpoB* from *M*. *parascrofulaceum*: the Type I genotype with the *M*. *parascrofulaceum rpoB* gene and the Type II genotype with the *M*. *intracellulare rpoB* gene. Additionally, we developed a novel SNP-based phylogenetic analysis to enable the taxonomic identification of *M*. *yongonense* clinical strains.

## Supporting Information

S1 Table Strains used in this study. (XLSX)
